# Blockade of the acute activation of mTOR complex 1 decreases hypertrophy development in rats with severe aortic valve regurgitation

**DOI:** 10.1186/s40064-015-1230-1

**Published:** 2015-08-20

**Authors:** Marie-Claude Drolet, Vincent Desbiens-Brassard, Elise Roussel, Veronique Tu, Jacques Couet, Marie Arsenault

**Affiliations:** Groupe de Recherche sur les Valvulopathies, Centre de Recherche de l’Institut universitaire de Cardiologie et pneumologie de Québec, Université Laval, 2725, Chemin Sainte-Foy, Quebec, QC G1V 4G5 Canada

**Keywords:** Heart, Aortic valve, Hypertrophy, Left ventricle, Aortic regurgitation, Volume overload, Rapamycin, mTOR

## Abstract

**Background:**

Hypertrophy (H) is an adaptive response of the heart to a hemodynamic overload. Severe left ventricular (LV) volume overload (VO) from valve regurgitations (aortic (AR) or mitral regurgitation) leads to eccentric LVH. Increased protein turnover is a major event during development of LVH and the mechanistic target of rapamycin (mTOR) is a key molecule for its control. The role of mTOR inhibition in the development of LVH using rapamycin for relatively short periods of time (days to a few weeks) has been studied in the past in pressure overload models but not in VO models. We investigated if mTOR pathway was activated during LVH development in a model of severe VO (AR) in rats and if a rapamycin treatment can slow heart remodeling in this situation.

**Methods and Results:**

Male rats with severe AR were studied acutely at 2 days, at 8 weeks (compensated phase) and 6 months (late phase) after VO induction. mTOR complex (mTORC) 1 (ribosomal S6 protein phosphorylation) was activated early after AR induction but not later in the disease whereas mTORC2 activity levels (Akt phosphorylation at Ser473) remained stable. We observed that a moderate dose of rapamycin (2 mg/kg/day; orally) for 8 weeks prevented severe LVH caused by AR (−46 %: p < 0.001). Rapamycin treatment specifically inhibited LV mTORC1 without altering mTORC2 activity at 8 weeks. Rapamycin also prevented cardiac myocyte hypertrophy caused by AR.

**Conclusion:**

Rapamycin slows hypertrophy in LV VO by inhibiting early activation of mTORC1 without modulating mTORC2.

## Background

Chronic aortic valve regurgitation (AR) will slowly alter left ventricular LV morphology and function. The remodeling associated with AR is characterized by dilation, eccentric hypertrophy and eventually diastolic and systolic heart failure (HF). The management of patients with heart valve regurgitation is currently limited to surgical valve replacement (Bekeredjian and Grayburn 2005). There is no approved medical treatment. In a rat model of severe and chronic AR, we have showed that low doses of beta-blockers can improve systolic function, reduce LV hypertrophy (LVH) and improve survival (Zendaoui et al. 2011; Champetier et al. 2009; Plante et al. 2004b, 2008). We also observed similar benefits by targeting the renin-angiotensin-aldosterone system in this model (Arsenault et al. 2013; Plante et al. 2004a, 2009; Couet et al. 2006; Zendaoui et al. 2012). However, the sequence of signaling events taking place in the development of eccentric LVH is not well understood.

Among the many signaling molecules acting downstream of the adrenergic and angiotensin II receptors, mechanistic target of rapamycin (mTOR) activation by a phosphoinositide-3-kinase (PI3K)-dependent and/or independent mechanism is often present (Bishu et al. 2013). Mechanical stress can also activate mTOR via the integrins-focal adhesion kinase (FAK) pathway in pressure overload (PO) LVH(Clemente et al. 2012). At least, two mTOR complexes (mTORC1 and mTORC2) exist and can modulate pathways that regulate translation, cell size, survival as well as autophagy (Laplante and Sabatini 2012, 2013). Activation of mTORC1 is responsible for increased protein synthesis. Its inhibition using rapamycin has been shown to decrease development of LVH and to induce its regression in PO animal models (Gao et al. 2006; McMullen et al. 2004; Shioi et al. 2003; Soesanto et al. 2009). mTORC2 activity has received less attention in the context of pathological heart hypertrophy (Volkers et al. 2013a; b). The mTORC2 complex when activated leads to the phosphorylation of Akt (protein kinase B) on serine 473 (S473) and to improved cell survival. mTORC2 activation has been shown recently to be cardioprotective in the context of myocardial ischemia (Volkers et al. 2013a). The mTORC2 complex is believed to be rapamycin-insensitive although demonstrations of rapamycin inhibition have been reported (Zeng et al. 2007; Sarbassov et al. 2006; Ye et al. 2012).

The general state of activation of mTOR signaling has not been studied in LV volume overload (VO) models causing eccentric hypertrophy. This is also true for the effects of rapamycin inhibition of mTOR in the context of eccentric LVH. We therefore designed a study to evaluate mTORC1 and mTORC2 activity in LV VO hypertrophy acutely (48 h post-AR), during the compensated phase of the disease (8 weeks) and at a later stage (6 months). We hypothesized that blocking protein translation from mTORC1 activation using rapamycin would slow LVH development in a model of severe VO from chronic AR. Severe AR is associated with significant diastolic LV stretch from increased preload caused by the regurgitating blood. This mechanical stress on the myocardium is an important trigger of LVH. We therefore also studied the state of mTOR activation during a mechanical stress (cyclic stretching) imposed to cardiac myocytes in culture in order to investigate the role of this pathway in initiating eccentric LVH.

## Methods

### Animal model of aortic regurgitation

Forty male Wistar rats (250–300 g, Charles River, QC, Canada) were divided in four groups. Severe AR was induced by retrograde puncture of the aortic valve leaflets as previously described (Plante et al. 2003; Arsenault et al. 2002) and AR rats randomly divided in 2 groups (n = 10–12/gr): untreated or treated with rapamycin 2 mg/kg/day, orally. Sham operated rats were used as controls and randomly assigned to similar groups: untreated or rapamycin. Rapamycin treatment was initiated one week before surgery and continued for a total of 8 weeks. In a second protocol, 50 untreated rats were divided into four groups: sham operated (n = 20) and AR (n = 30). Ten animals per group were euthanatized 2 days later and the rest after 6 months. At the end of the protocol, hearts were quickly dissected, weighed and processed for further analysis.

### Echocardiography

A complete M-Mode, 2D and Doppler echocardiogram was performed on the animals under 2.5 % inhaled isoflurane anesthesia using a 12 MHz probe with a HD11XE echograph (Philips Medical Imaging, Andover, MA) immediately before, during surgery and at the end of protocol (Lachance et al. 2009).

### Cell culture studies

HL-1 cells (a murine atrial cardiomyocyte cell line) were cultured in fibronectin (0.5 %, w/v)/gelatin (0.02 %, w/v) (Sigma)-coated flasks as described (Claycomb et al. 1998). Cells were cultured in Claycomb medium (Sigma Aldrich, Mississauga, Ont, Canada) containing 10 % (v/v) fetal bovine serum (Sigma-Aldrich), 0.1 mM norepinephrine (Sigma), 2 mM l-glutamine (Sigma), 100 IU/ml penicillin and 100 μg/ml streptomycin (Invitrogen, Burlington, ON, Canada) and maintained at 37 °C under 5 % CO2.

### Mechanical stretch protocols

HL-1 cells were subjected to equi-biaxial mechanical stretch using a Flexcell FX 5000 Tension System (Flexcell, Hillsborough, NC). Cells were seeded at 10^6^ cells/well density in 6-well BioFlex culture plates coated with fibronectin (0.5 %, w/v)/gelatin (0.02 %, w/v). The day after, cells were serum-starved for 4 h then subjected to cyclic stretch to produce an 18-20 % elongation at 1 Hz. Cells not subjected to mechanical stretch were used as controls.

### Analysis of mRNA accumulation by quantitative RT-PCR

The analysis of LV mRNA levels by quantitative RT-PCR has been described in details elsewhere (Champetier et al. 2009).

### Immunoblotting

Crude LV or HL-1 homogenates were separated by SDS-PAGE. All primary antibodies were used at a 1:1000 dilution and were purchased from Cell Signaling Technology (Beverly, MA) or from Santa Cruz Biotechnology (Santa Cruz, CA) as described elsewhere (6).

### Statistical analysis

Results are presented as mean ± SEM. Intergroup comparisons were done using 2-way ANOVA followed by Bonferroni post-test if interaction between disease (AR) and treatment (Rapa) was significant. One-way ANOVA followed by Tukey’s post-test was used for culture cells studies. Student t test was used when two groups were compared directly. Statistical significance was set at p values <0.05. Data and statistical analysis were performed using Graph Pad Prism version 6.02 for Windows, Graph Pad Software (San Diego CA).

## Results

### Rapamycin inhibits LV eccentric hypertrophy caused by AR

**Fig. 1:**
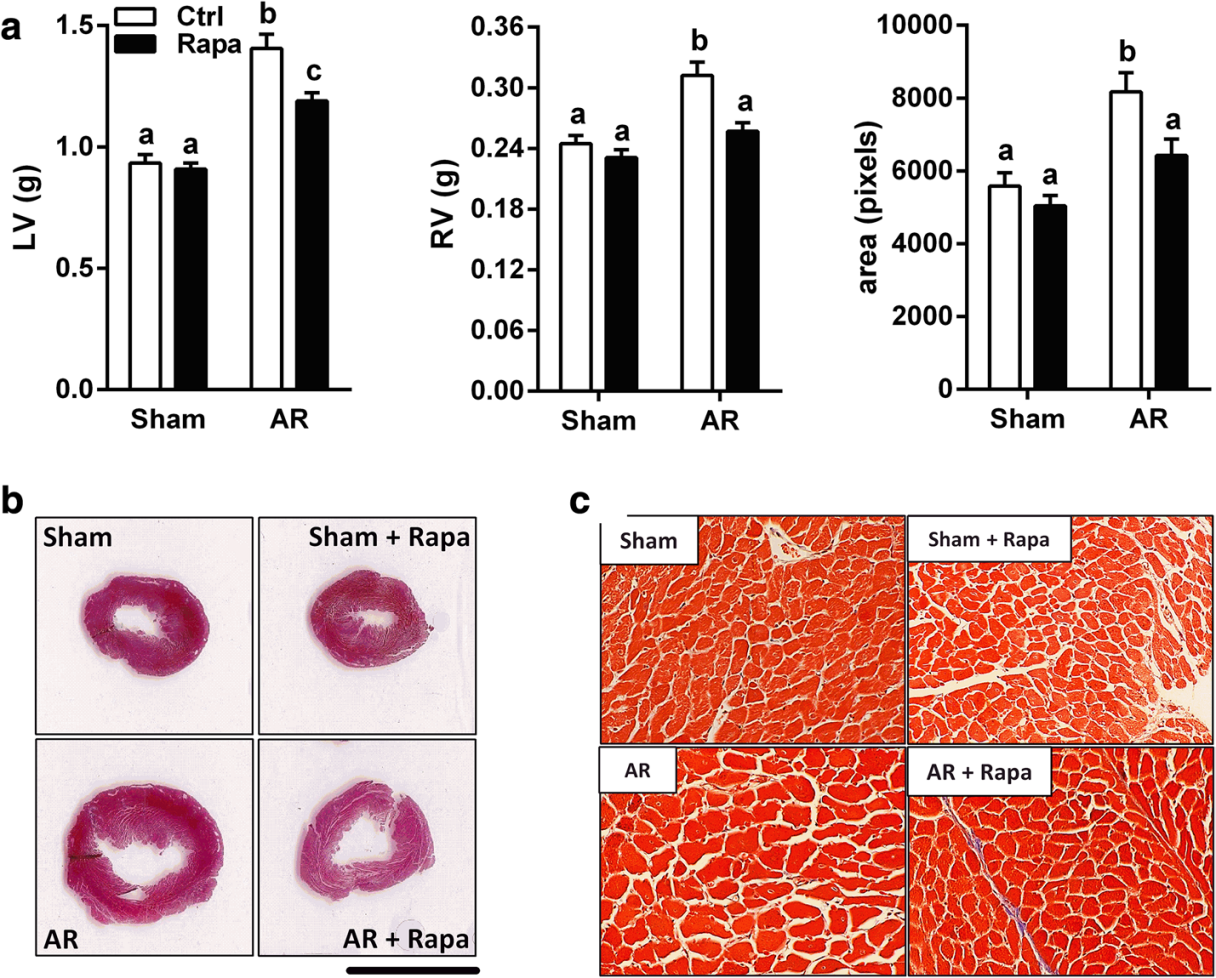
Rapamycin reduces LV and RV hypertrophy and cardiac myocytes hypertrophy in AR rats. **a** LV and right ventricle (RV) weight after 8-week VO from AR and LV cardiac myocytes cross-sectional area (CSA) evaluated in mid-LV sections stained with Trichrome-Masson. **b**, **c** Representative Trichrome-Masson LV sections at different magnifications (**b**: *bar* = 1 cm and **c**: X200). For CSA, three sections per animals were analyzed and at least 20 myocytes per section measured by an observer blinded for the groups. n = 9–12. Means not sharing a common superscript are significantly different from each other, P < 0.05

AR caused severe heart hypertrophy. Rapamycin treatment significantly reduced LVH in AR rats (Table 1). Rapamycin treatment had no impact on heart weight in sham-operated animals. A similar pattern was observed also for both left and the right ventricles in AR animals where rapamycin inhibited hypertrophy by 41 and 49 %, respectively (p < 0.01 for both) (Fig. 1a, b). Myocytes cross-sectional area was larger in the untreated AR group compared to healthy rats (+46 %; p < 0.001). Rapamycin reversed this increase by 67 % (p < 0.05; Fig. 1a, c). We did not notice any significant accumulation of interstitial or perivascular fibrosis in AR animals at 8 weeks (Fig. 1c). As expected and previously reported by others, body weight was lower in the animals treated with rapamycin probably related with decreased food intake (34 g/day, untreated vs. 28, rapa; p = 0.03) leading to a lower adipose tissue mass (Festuccia et al. 2014; Blanchard et al. 2012). Tibial length (an index of body growth) was only slightly reduced (1 %; p = 0.006) by rapamycin suggesting that treated animals were just leaner (Table 1). Echocardiographic data at the end of the protocol are also summarized in Table 1. As expected, AR resulted in significant end-diastolic and end-systolic LV dilatation. Ejection fraction was lower in AR animals. Rapamycin treatment in AR significantly reduced end-diastolic and end-systolic diameters compared to untreated AR. AR severity was similar in both AR groups.

**Table 1:** Animal characteristics and echocardiography data at the end of the protocol

Parameters	Sham (n = 10)	Sham Rapa (n = 9)	AR (n = 12)	AR Rapa (n = 10)
Heart (mg)	1285 ± 45^a^	1245 ± 31^a^	1903 ± 63^b^	1577 ± 52^c^
Tibial length (mm)	57 ± 0.3^a^	56 ± 0.4^b^	58 ± 0.5^a^	56 ± 0.3^b^
Ind. Heart (mg/mm)	2.3 ± 0.07^a^	2.2 ± 0.05^a^	3.3 ± 0.09^b^	2.8 ± 0.10^c^
Left atria (mg)	29 ± 1.5^a^	36 ± 2.1^a^	55 ± 4.7^b^	51 ± 5.9^b^
Lungs (g)	2.9 ± 0.5^a^	3.1 ± 0.5^a^	3.4 ± 0.4^a^	3.2 ± 0.4^a^
Body weight (g)	564 ± 11.6^a^	491 ± 11.3^b^	562 ± 10.2^a^	474 ± 14.4^b^
EDD (mm)	8.6 ± 0.17^a^	8.4 ± 0.16^a^	11.0 ± 0.16^b^	9.8 ± 0.12^c^
ESD (mm)	4.1 ± 0.15^a^	3.8 ± 0.18^a^	6.3 ± 0.22^b^	5.8 ± 0.20^b^
SW (mm)	1.3 ± 0.04^a^	1.4 ± 0.07^a^	1.8 ± 0.05^b^	1.6 ± 0.05^c^
PW (mm)	1.4 ± 0.06^a^	1.4 ± 0.06^a^	1.8 ± 0.05^b^	1.7 ± 0.08^b^
EF (%)	78 ± 1.5^a^	79 ± 1.8^a^	68 ± 1.9^b^	66 ± 1.6^b^
AR (% reg.)	na	na	75 ± 4^a^	71 ± 3^a^

Number of animals per group in parentheses. Values are expressed as mean ± SEM. Means not sharing a common superscript are significantly different from each other, P < 0.05. *Sham* sham-operated animals and *Rapa* rapamycin. *EDD*: end-diastolic diameter, *ESD* end-systolic diameter, *SW* septal wall thickness, *PW* posterior wall thickness, *EF* ejection fraction, *AR* AR severity by echocardiographic semi-quantification and *na* non applicable

### Rapamycin inhibition of mTORC1 but not of mTORC2 in rat LVs

**Fig. 2:**
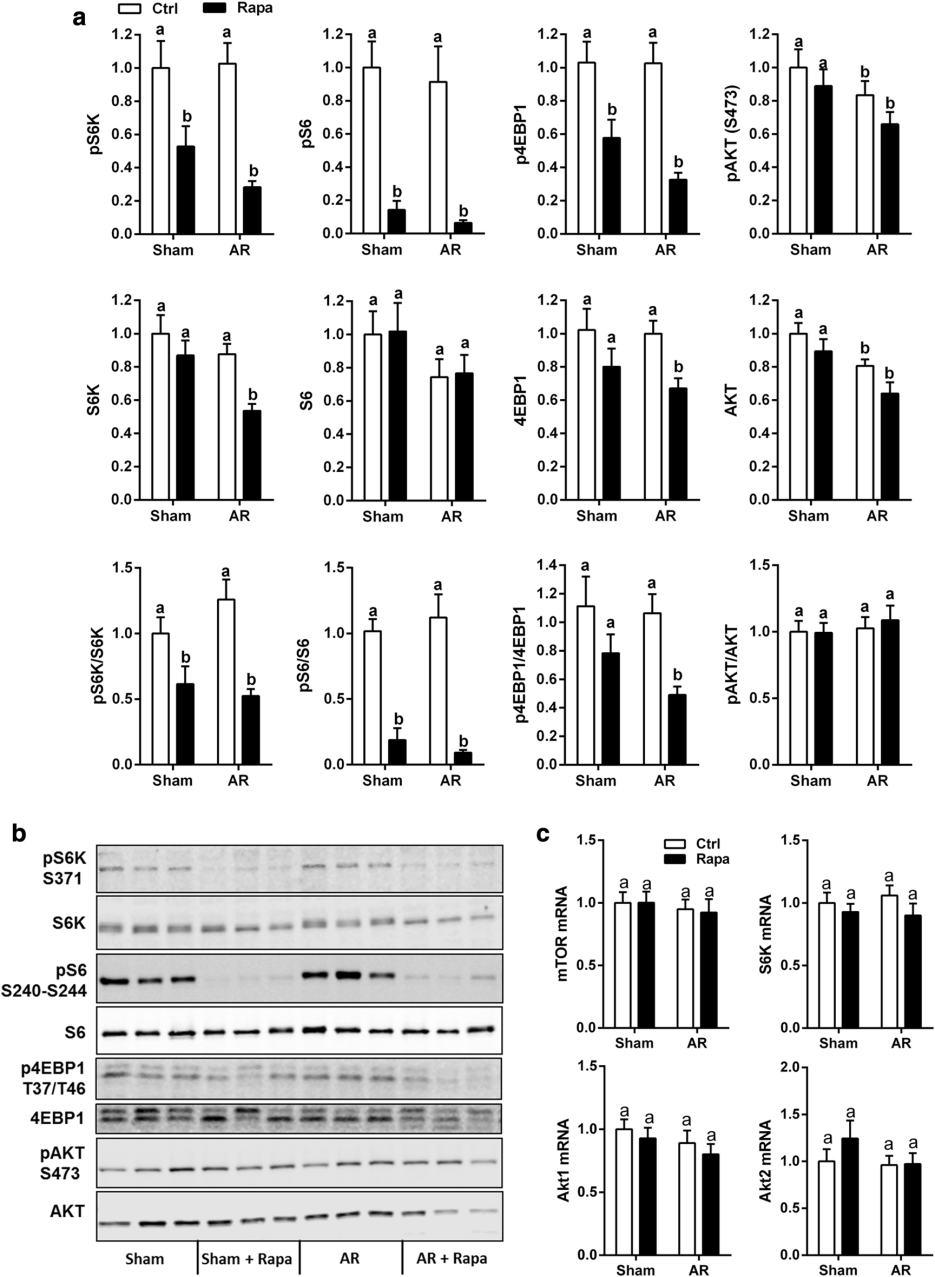
Rapamycin inhibits mTORC1 but not mTORC2 LV activity in AR rats. LV protein content of total and phosphorylated forms of Akt, S6 kinase (S6K), S6 and 4EBP-1 in sham and AR rats treated or not with rapamycin. **a**, **b** Immunoblotting. **c** Gene expression of mTOR, S6 Kinase and Akt1 and Akt2. n = 9–12. Means not sharing a common superscript are significantly different from each other, P < 0.05

Chronic rapamycin treatment has been shown to inhibit not only the activity of the mTORC1 but also of mTORC2 by binding free mTOR and blocking complex formation (Sarbassov et al. 2006). We measured the protein content of four proteins implicated in mTOR signaling namely p70S6 kinase (S6 K), S6, 4EBP1 and Akt. AR did not lead to increased phosphorylation levels of those signaling proteins compared to sham animals at 8 weeks (Fig. 2a, b). On the other hand, rapamycin treatment decreased the protein content ratio of phospho (p)-S6K/S6K, pS6/S6 and p4EBP1/4EBP1 while pAKT (S473)/AKT levels remained stable. We observed in AR and AR + Rapa groups slightly lower levels of the total forms of these signaling molecules suggesting that LV VO could affect their expression. We thus evaluated mTOR, S6K and Akt1 and Akt2 gene expression in the LV of the experimental groups. As illustrated in Fig. 2c, mRNA levels for these four genes remained stable in all groups.

### Hypertrophy and extracellular matrix remodeling markers

**Fig. 3:**
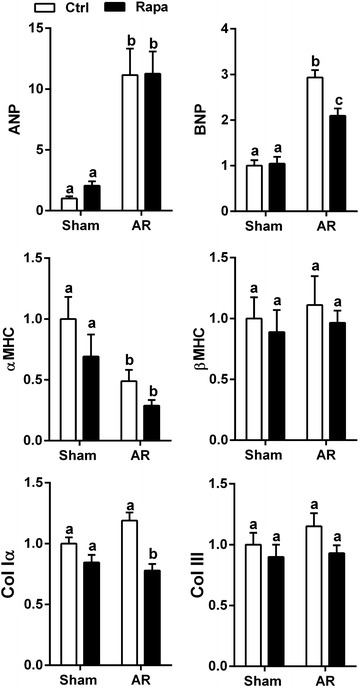
Hypertrophic markers expression in AR rats. LV mRNA levels of natriuretic peptides (ANP and BNP; *top row*), myosin heavy chains (α and β; *middle row*) and collagens (Type I and III, *bottom row*) were evaluated as described in “Methods” section. *White columns*: untreated groups (*ctrl*); *black columns*: rapamycin groups (*rapa*). n = 9–12. Means not sharing a common superscript are significantly different from each other, P < 0.05

The relative expression of ANP and BNP mRNAs were measured in LV tissues. Results are reported in Fig. 3. All AR groups at 8 weeks displayed a significant increase in ANP and BNP mRNA expression. BNP mRNA levels were significantly decreased by rapamycin treatment in AR animals. The same was true for the pro-hypertrophic TRPC6 gene (not shown). Myosin heavy chains (MHC) mRNA levels were also measured. The αMHC mRNA levels were lower in the AR groups whereas βMHC mRNA levels remained stable. Rapamycin did not significantly affect these levels. Collagen type I mRNA levels were higher in untreated AR animals but rapamycin reversed this increase. Collagen type III mRNA levels were not regulated.

### mTORC1 but not mTORC2 is transiently activated in VO LVH

**Fig. 4:**
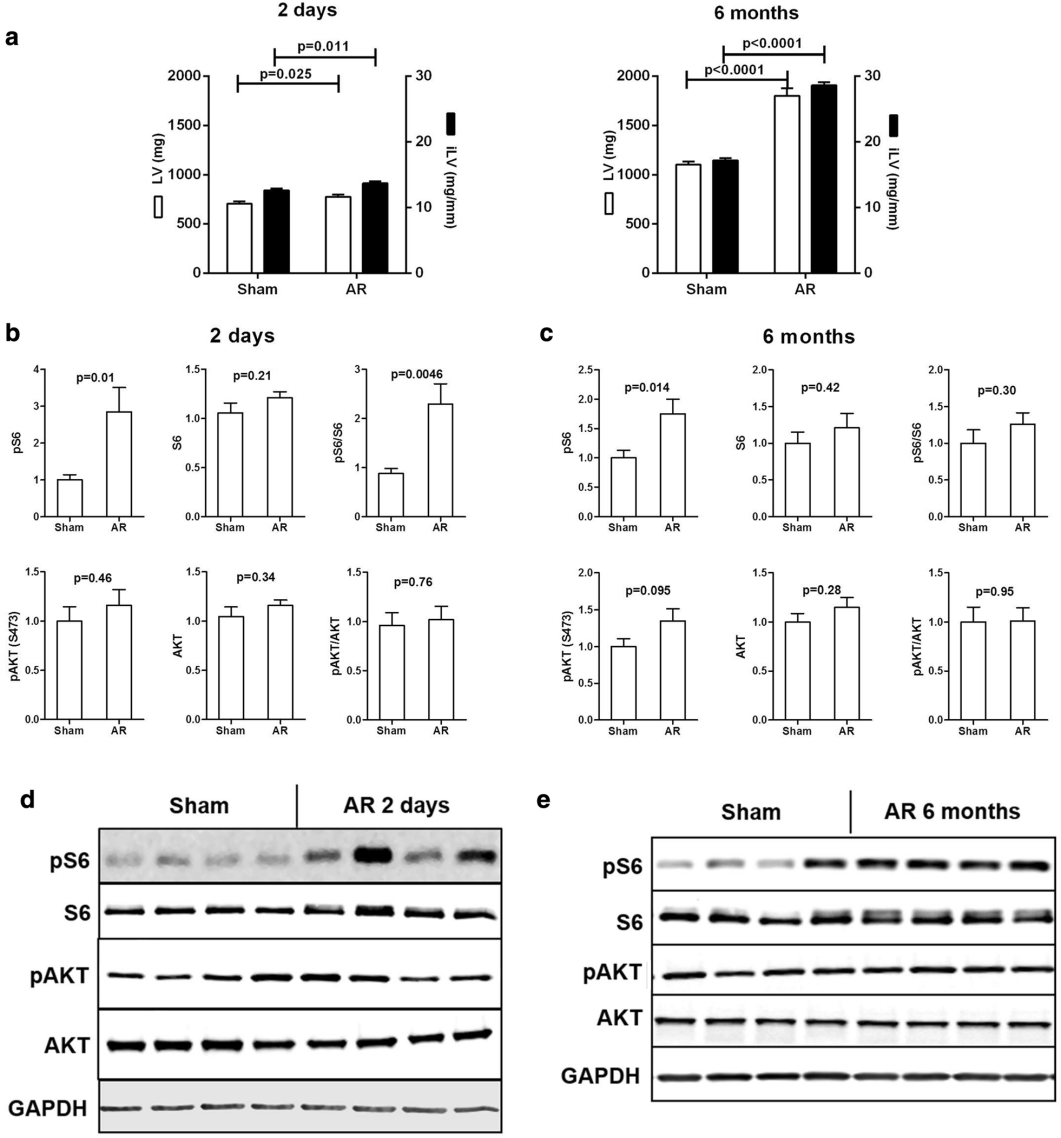
mTORC1 is transiently activated in LV VO. Protein content of the phosphorylated (p) and total forms of S6 and Akt (Ser473) in the LV of AR rats two days and 6 months post-surgery. **a** Characteristics of AR rats after 2 days or 6 months. **b**, **c** LV protein contents of 2-Day and 6-month AR rats. **d**, **e** Representative blots. n = 9–12. Means not sharing a common superscript are significantly different from each other, P < 0.05

Since AR did not seem to modulate the different members of the mTOR signaling pathway studied at 8 weeks, we measured in LV crude homogenates the contents of ribosomal S6 protein as an index of mTORC1 activity and AKT (S473) as an index of mTORC2 activity at other stages of the disease (Volkers et al. 2013a). Two days of severe volume overload (>70 % regurgitation) were sufficient to initiate a hypertrophic response in AR rats as illustrated by the small but significant increase in LV weight (Fig. 4a). S6 phosphorylation was strongly increased in the LVs of AR rats 2 days post-AR induction while pAKT levels remained stable (Fig. 4b, d). A trend for a similar pattern was also present 6 month post-AR in the LV of surviving animals (15 out of 20) although the pS6/S6 ratio remained unchanged (Fig. 4c, e).

### Cardiac myocytes mechanical stretch activated mTORC1 and mTORC2

**Fig. 5:**
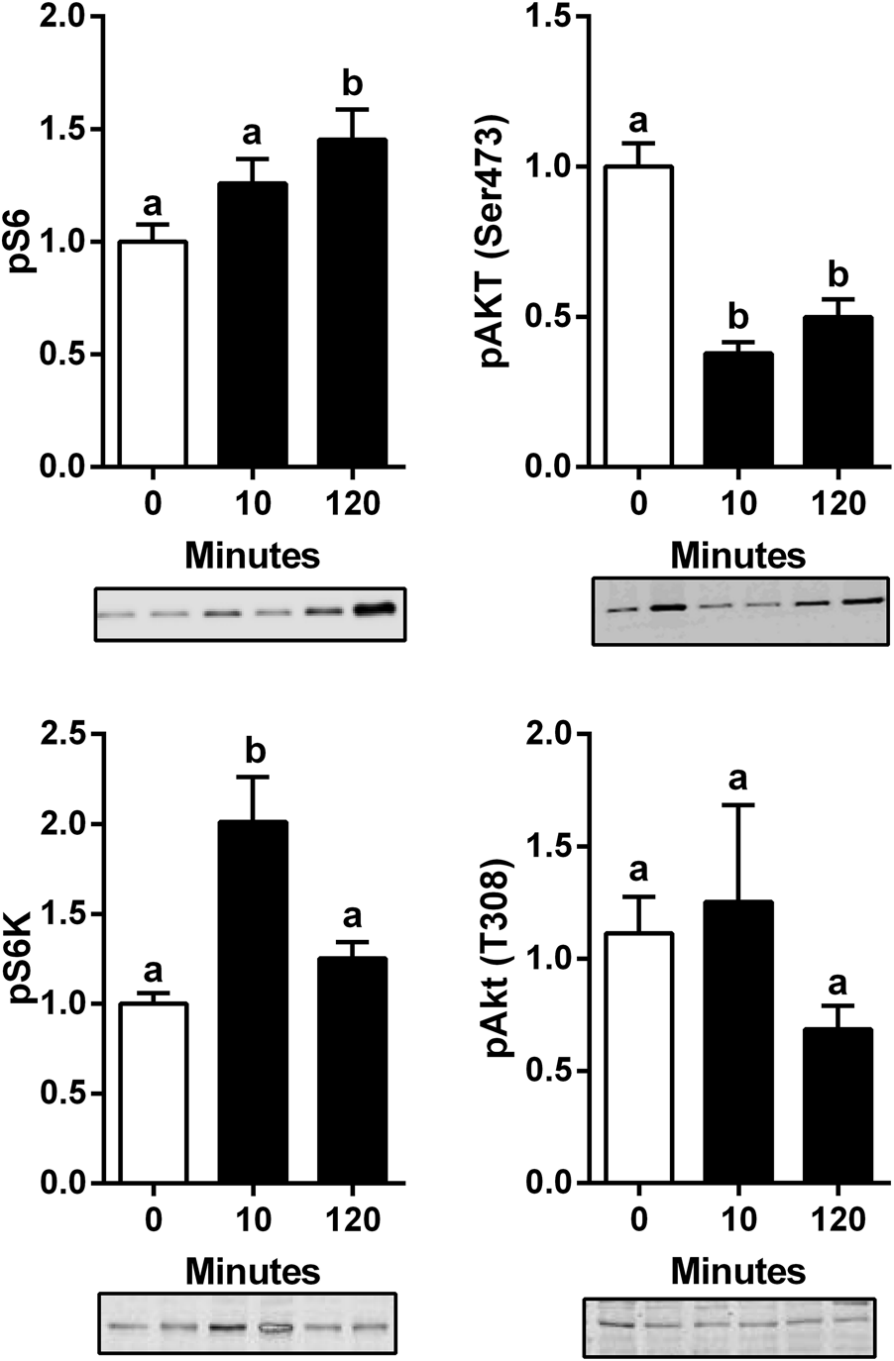
Mechanical stretch activates mTORC1 in HL-1 cells. Protein contents of the phosphorylated forms of Akt (Ser473 and Thr308), K and S6 in HL-1 cells mechanically stretched for the indicated time. Cells were serum-starved for 4 h then incubated in DMEM supplemented with 0.1 % bovine fetal serum during cyclic stretch. n = 6. Means not sharing a common superscript are significantly different from each other, P < 0.05. Representative blots below graphs

Severe AR results in important diastolic wall stress and cardiac myocytes stretch. In order to reproduce this type of mechanical stress in vitro, HL-1 cells were submitted to cyclic mechanical stretching before incubation with insulin for 30 min. The phosphorylation levels of various mTOR-related proteins were then measured by immunoblotting (Fig. 5). When a mechanical stretch stress was applied to the cells, S6 kinase and S6 phosphorylation levels increased after 10 and 120 min, respectively. Akt phosphorylation levels on Thr308 remained unchanged while those of S473 were reduced.

### Transient FAK activation in AR LVs

**Fig. 6:**
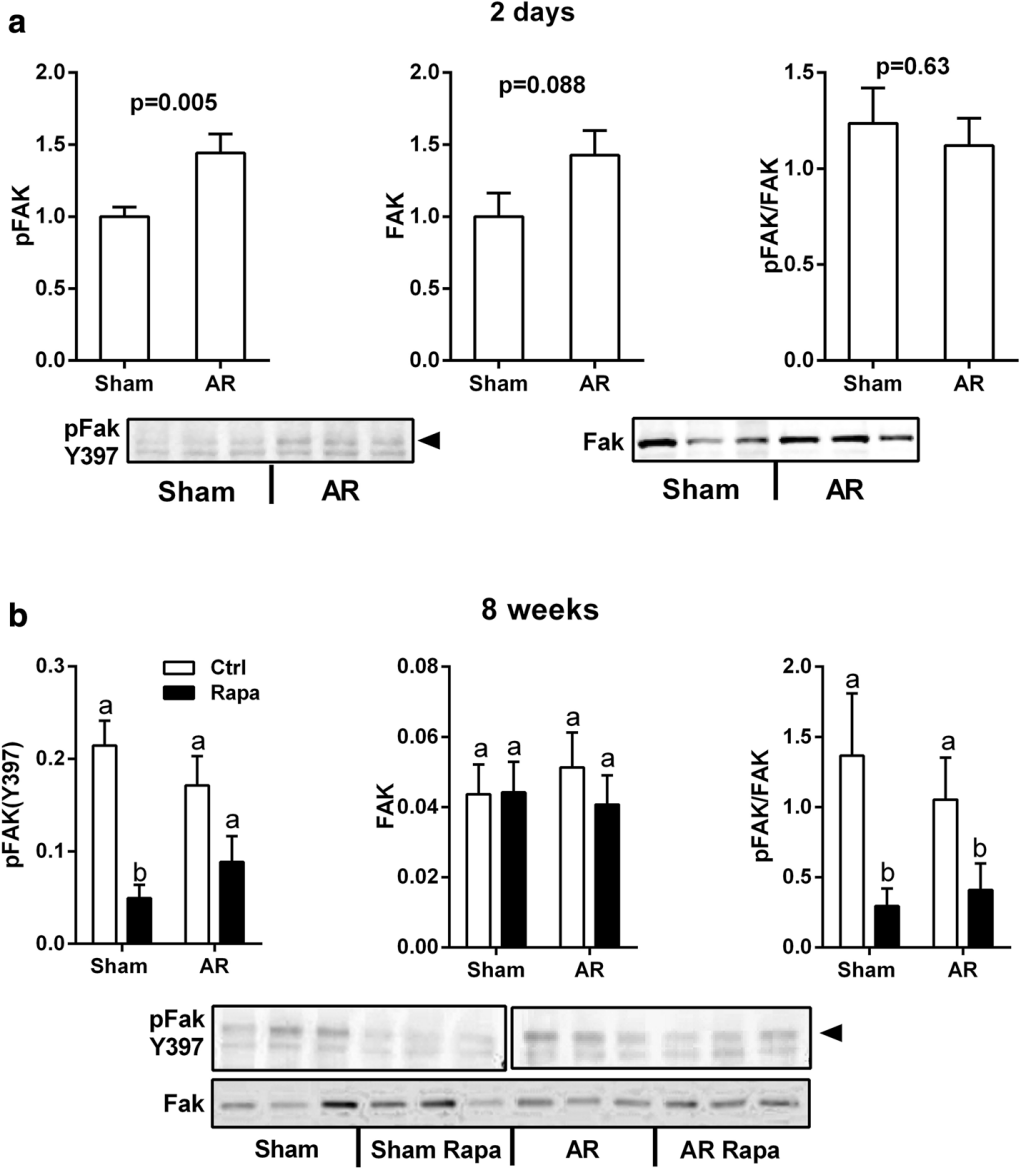
Focal adhesion kinase (FAK) activation in LV VO. Protein contents of the phosphorylated and total forms of FAK (Y397) were evaluated by immunoblotting in the left ventricles of AR rats two days (**a**) and 8 weeks (**b**) post-surgery. n = 8. Means not sharing a common superscript are significantly different from each other, P < 0.05. Representative blots below graphs. The 8-week pFAK blot is presented in two different *panels* as a molecular weight marker was cropped from the image between Sham and AR samples

Focal adhesion kinase (FAK) is implicated in the signal transduction of mechanical stress. Two days post-AR, both FAK and pFAK were increased in the LV (Fig. 6a) while it was not the case later at 8 weeks (Fig. 6b). Rapamycin treatment decreased FAK phosphorylation in both sham-operated and AR animals. We also measured the pFAK(Y397)/FAK ratio in stretched HL-1 cells. We observed a strong trend for an increase of FAK phosphorylation (+70 %; p = 0.055) (not shown).

### Autophagy markers in the AR myocardium

**Fig. 7:**
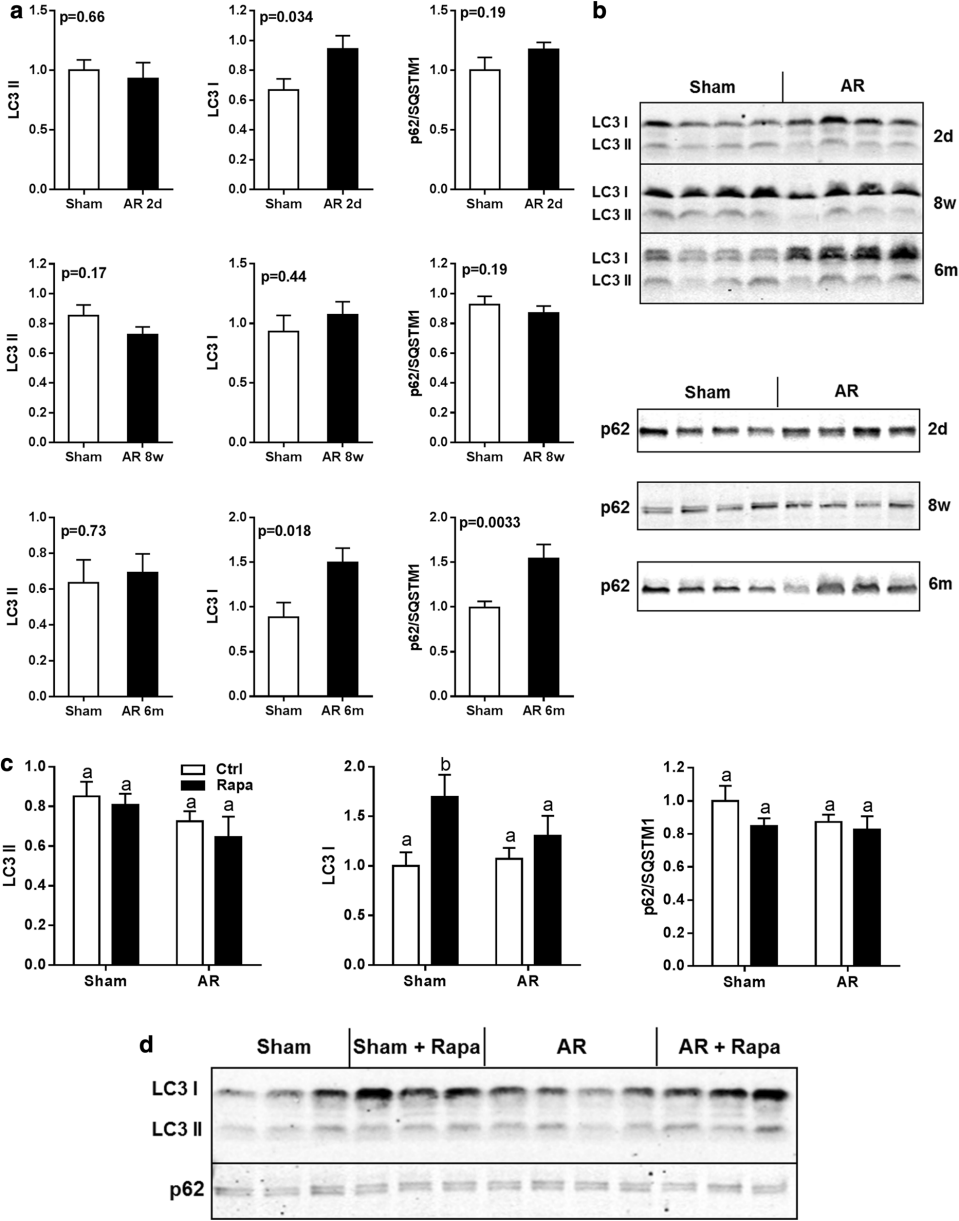
Autophagy markers in LV VO. LC3 and p62 protein contents were evaluated at various times post-AR induction (**a**, **b**) as well as the impact of rapamycin treatment (**c**, **d**). N = 9–12 animals per group. Values are expressed as mean ± SEM. Means not sharing a common superscript are significantly different from each other, P < 0.05

Autophagy can help eliminate damaged proteins and help survival in cardiac hypertrophy but excessive autophagy may lead to heart failure. mTORC1 is a known regulator of autophagy and we were interested to see if the acute mTORC1 activation in AR LVs could influence this process. We studied the levels of protein markers associated with autophagy namely LC3 and p62/sequestrin. As illustrated in Fig. 7, LV LC3 II levels remained unchanged at the various times post-AR studied. On the other hand, LC3 I levels were more elevated acutely after 2 days post-AR and later in the disease. Rapamycin treatment elevated LC3 I levels in the LVs of AR rats. Moreover, p62 levels were stable after 2 days and 8 weeks (treated or not with rapamycin) but were increased later in the disease.

## Discussion

We report that rapamycin can reduce LV dilation and cardiac myocyte hypertrophy caused by severe LV VO from AR. We observed that mTORC1 and mTORC2 activities were not increased in the myocardium during the compensated phase of the disease (8 weeks) but that mTORC1 activity was increased acutely 2 days after post-AR induction. The rapamycin dosage used in our study led to the inhibition of mTORC1 without altering LV mTORC2 activity. These findings are somewhat different from those of previous studies in PO LVH where mTORC1 activation was shown to be sustained over time (Shioi et al. 2003; McMullen et al. 2004; Ha et al. 2005; Gao et al. 2006; Bishu et al. 2013). We hypothesize that eccentric remodeling (dilation) after VO induction results in lowering of LV wall tension thus decreasing the mechanical stress responsible for mTORC1 activation. Interestingly, FAK activation also follows this pattern. This also suggests that the involvement of the mTOR pathway in the process leading to LVH induced by PO or VO is not completely similar.

### Rapamycin and mTORC2

The canonical target of rapamycin is mTOR. Rapamycin binds directly to FKBP12 and inhibits mTORC1 via a drug-induced interaction (Sabatini et al. 1994). The effects of chronic rapamycin exposition on mTORC2 formation have been previously explored in various cell lines as well as in mice treated for 1 week with 10 mg/kg intraperitoneally (IP) (Sarbassov et al. 2006). Interestingly, it was observed that rapamycin at this dosage reduced pAktS473 levels in the heart, an index of mTORC2 activation. We used a lower dose of rapamycin (5 times) and administered it orally. In spontaneously hypertensive rats, a 3-week rapamycin treatment (2 mg/kg/d) delivered by IP injection also led to decreased pAktS473 levels (Soesanto et al. 2009). Circulating rapamycin levels were likely much lower in our study. Recently, Bishu and al. measured serum rapamycin levels after administration of 2 mg/kg/day either IP or orally in mice and showed that the oral route led to levels corresponding to a third of those from the IP administration route (Bishu et al. 2013). In a study on kidney transplant patients studying the impact on LVH of rapamycin treatment, the authors found benefits after switching patient from cyclosporine to rapamycin (Paoletti et al. 2008). Rapamycin serum levels were titrated to be maintained between 5 and 15 ng/ml levels similar to those measured in the study of Bishu et al. in mice (Bishu et al. 2013). Our rapamycin treatment was sufficient to reduce LVH by more than 40 % in AR rats without slowing normal heart growth that still takes place in rats this age. This supports the idea that relatively low-dose of rapamycin may provide benefits by inhibiting mTORC1 without affecting the cardio-protective mTORC2.

### mTOR inhibition and the heart

Pharmacological mTOR inhibition by rapamycin has been shown to improve cardiac function in PO LVH, after myocardial infarct, in a LEOPARD syndrome model and after kidney transplantation (Gao et al. 2006; Kuzman et al. 2007; Shioi et al. 2003; Marin et al. 2011). On the other hand, mTOR signaling is essential for normal heart development and physiology (Shen et al. 2008; Zhu et al. 2013). Ablation of mTOR in cardiac myocytes rapidly induces heart failure (HF) in adult mice (Zhang et al. 2010). A similar phenotype is observed in mice lacking cardiac Raptor (mTORC1) (Shende et al. 2011) whereas postnatal rictor (mTORC2) deletion in cardiac myocytes does not lead to an observable abnormal phenotype. However, under PO, these mice evolved more quickly towards heart failure than wild type mice (Volkers et al. 2013a). It is somewhat of a paradox that genetic ablation of mTOR or of its two main binding partners in the heart leads to adverse phenotypes whereas mTOR inhibition with rapamycin provides benefits in the overloaded heart. We observed that rapamycin reduced LVH development by limiting cardiac myocytes hypertrophy in AR rats suggesting that mTORC1 inhibition might protect the heart under VO.

### mTOR, FAK and LV wall stress in VO

Recent studies have suggested that Akt and mTOR are downstream effectors of pro-hypertrophic signaling mediated by the focal adhesion kinase (FAK) (Clemente et al. 2012). Our results in stretched cardiac myocytes also support the idea that mechanical stretch leads to the activation of FAK. Rapamycin has been shown to suppress cardiac myocytes hypertrophy when submitted to cyclic stretch (Marin et al. 2008). AR results in an important increase of diastolic wall stress which in turn, leads to eccentric LV remodeling (Lachance et al. 2008). We evaluated how mechanical stretching would influence the mTOR pathway in HL-1 cells. Interestingly, we observed mTORC1 activation whereas mTORC2 was inhibited. The activation of mTORC1 could be the result of an integrin-FAK pathway in HL-1 cells. FAK activation can lead to mTORC1 activation coupling mechanical stress to a hypertrophic response (Clemente et al. 2012; Franchini 2012). Two days post-AR, pFAKs levels were increased along with a similar increase in total FAK content. At 8 weeks, this pFAK increase had disappeared suggesting that wall stress has been in part normalized by compensatory eccentric LV remodeling. We did not measure LV wall stress in our animals.

### mTOR activation in LV VO

The activation of mTORC1 and mTORC2 was not observed at 8 weeks post-AR. MTORC1 activation happened very quickly (2 days) after induction of severe LV VO. Our results at 8 weeks are in line with those we obtained in a recent study where we observed that S6K (mTORC1) and Akt S473 (mTORC2) levels were unchanged at 8 weeks in this model (Bouchard-Thomassin et al. 2011). At 6 months, we previously reported that Akt S473 phosphorylation was increased suggesting that mTORC2 becomes activated. This coincides with a decrease in animal survival along with a sharp increase in replacement fibrosis (Arsenault et al. 2013; Lachance et al. 2009). In this study, we did not observe this elevation in Akt phosphorylation by mTORC2 although a trend was present. The activation of mTORC2 at later stages is probably a response to a need for cell survival mechanisms in the dilated myocardium. At this late stage of the disease, survival rate is around 70 % and quickly falls afterwards suggesting that the myocardium is exhausting its capacity to assure its normal function (Plante et al. 2008; Lachance et al. 2009; Arsenault et al. 2013; Dhahri et al. 2014).

### Autophagy markers in VO

Activation of autophagy has been reported in the hypertrophied heart from PO (Sun et al. 2013) but had not been studied in VO hypertrophy. It is believed that autophagy can be beneficial during the initial myocardial remodeling phase while it can become detrimental during LV decompensation (Sciarretta et al. 2012; Lavandero et al. 2013). The markers we studied are not sufficient to entirely picture the autophagic processes that could be at play in the myocardium under VO. On the other hand, with the exception of up-regulation of LC3 I content acutely, during the late stage of the disease as well as by rapamycin, we did not record clear evidences that LC3 became more lipidated (LC3 II) at the various stages of the disease in AR animals, a feature of macroautophagy. On the other hand, an increase in the autophagic flux is often accompanied with accelerated turnover of LC3 II (Mizushima et al. 2010). The significance of an increase in the content of LC3 I is not clear but it can suggest that the cells may be more prone to undergo autophagy. If this is the case, mTOR inhibition by rapamycin in both sham and AR animals may stimulate autophagy which may be unwanted at least in normal animals. We did not study alternative autophagy pathways (atrogin5 and atrogin7-independent) and it cannot be excluded that they may take place in VO LVH (Shimizu et al. 2010). A study better designed to study the contribution of the autophagic processes in the development and evolution of eccentric LVH will be necessary to settle the issue.

### Systemic impact of rapamycin treatment

Rapamycin treatment significantly slowed body weight gain with little to no effect on body or heart growth. This decrease in body weight gain from rapamycin treatment had been observed before and it is believed to be the result of decreased food intake and particularly, of food efficiency (body weight gain vs. food intake) resulting in a decrease in adipose tissue mass (Festuccia et al. 2014; Blanchard et al. 2012). Rapamycin at higher dose that the one used has not been associated with muscle loss in rats (Deblon et al. 2012). Chronic rapamycin administration is associated with several metabolic imbalances such as increased glycaemia and insulin resistance (Houde et al. 2010; Veilleux et al. 2010) and thus, its long-term utilization in the treatment of cardiac diseases could be problematic. On the other hand, the use of moderate doses of this drug was sufficient to slow LVH development in our model suggesting that a possible therapeutic window may exist for developing drugs targeting mTORC1.

## Conclusion

In conclusion, we report that mTORC1 is transiently activated after induction of severe LV VO from aortic valve regurgitation and that rapamycin inhibition can partially block LVH development during eccentric remodeling. On the other hand, chronic rapamycin treatment (8 weeks) at the dose of 2 mg/kg/day orally was not associated with mTORC2 inhibition. Our results also suggest that mTORC1 becomes activated following a mechanical stress in cardiac myocytes and that activation probably decreases when wall stress is normalized after LV dilation Our findings open the door to potential new therapeutic molecular targets in LV VO that deserves further investigation.
